# Articulation posture influences pitch during singing imagery

**DOI:** 10.3758/s13423-023-02306-1

**Published:** 2023-05-23

**Authors:** Anita Körner, Fritz Strack

**Affiliations:** 1 Department of Psychology, University of Kassel, Holländische Straße 36–38, 34127 Kassel, Germany; 2 Department of Psychology, University of Würzburg, Würzburg, Germany

**Keywords:** Singing imagery, Pitch, Musical imagery, Language, Embodied cognition

## Abstract

Facial muscle activity contributes to singing and to articulation: in articulation, mouth shape can alter vowel identity; and in singing, facial movement correlates with pitch changes. Here, we examine whether mouth posture causally influences pitch during singing imagery. Based on perception–action theories and embodied cognition theories, we predict that mouth posture influences pitch judgments even when no overt utterances are produced. In two experiments (total *N* = 160), mouth posture was manipulated to resemble the articulation of either /i/ (as in English *meet;* retracted lips) or /o/ (as in French *rose;* protruded lips). Holding this mouth posture, participants were instructed to mentally “sing” given songs (which were all positive in valence) while listening with their inner ear and, afterwards, to assess the pitch of their mental chant. As predicted, compared to the o-posture, the i-posture led to higher pitch in mental singing. Thus, bodily states can shape experiential qualities, such as pitch, during imagery. This extends embodied music cognition and demonstrates a new link between language and music.

**Supplementary Information:**

The online version contains supplementary material available at 10.3758/s13423-023-02306-1.

## Introduction

The muscles that shape the mouth have several functions, prominently among them articulating speech and expressing emotions. Accordingly, changing mouth posture, specifically, lip corner retraction (vs. lip rounding) has been found to influence mood (Coles et al., 2022) and acoustic properties of articulated vowels (Fagel, 2010). Connecting emotional and articulatory properties, articulation has been found to be associated with word valence, such that words whose vowels require lip retraction were associated with more positive valence than words whose vowels require lip rounding (Körner & Rummer, 2022a). In the present research, we extend the research concerning the influences of mouth posture to pitch, examining whether mouth posture causally influences pitch during mental singing.

## Pitch in singing

Vocal sounds are produced by air flow passing from the lungs through the vocal tract. Sound waves are produced in the larynx and then modulated by other articulators before they are emitted. The major determinant of pitch is the vibration speed of the vocal folds, while the configuration of later articulators, for example, tongue and jaw position, mainly influence other aspects of the vocal product, such as vowel formants and breathiness of the voice (e.g., Titze, 1994; Wolfe et al., 2020). Thus, physiologically speaking, mouth posture is not a major determinant of pitch. Accordingly, trained singers are able to vary pitch independent of facial posture (e.g., Lange et al., 2022). Nevertheless, initial evidence suggests that facial movement during singing correlates with pitch production and perception. When singing, facial movement of the singer varies with interval size, so that head movement, eyebrow movement, and lip movement increase with increasing interval size (Thompson & Russo, 2007). Moreover, other people use these cues when predicting sung pitch. Thus, singers’ facial muscle activity influences pitch judgment in listeners (Laeng et al., 2021; Thompson et al., 2010; Thompson & Russo, 2007). The present research directly manipulates facial muscle activity – in a way unrelated to the singing task – and asks singers to evaluate pitch in mental singing.

Mental singing refers to an imagery process where one’s inner voice (i.e., a covert articulation mechanism) creates a chant that can be perceived with one’s inner ear (i.e., an imagery of hearing speech) – without overt utterances (for reviews, see Hubbard, 2010, 2019; Perrone-Bertolotti et al., 2014). Auditory imagery in general is highly accurate, resembling the perception of overt stimuli, for example, in duration, loudness, and pitch (e.g., Farah & Smith, 1983; Halpern, 1988; Wu et al., 2011). Mental singing involves a covert activation of articulation muscles (Smith et al., 1995), leading to increased larynx activation during mental singing (vs. listening to music; Bruder & Wöllner, 2021; see also Pruitt et al., 2019). Moreover, articulation suppression has been found to reduce involuntary song imagery (Beaman et al., 2015) and to interfere with the memory for songs (Wood et al., 2020; cf. Weiss et al., 2021). Thus, covert articulation can be assumed to causally contribute to mental singing, influencing auditory imagery.

## Perception–action links

The influence of covert articulation on auditory imagery can be explained by action–perception theories (e.g., Hommel, 2019; Knoblich & Sebanz, 2006; Shin et al., 2010; Witt, 2011). According to action–perception theories, action planning involves predicting the anticipated sensory consequences of the action, so that perception and action involve shared processes and shared representations. Specifically, for speech perception and production, the motor theory of speech perception (Galantucci et al., 2006; Liberman & Mattingly, 1985) postulates that perceiving speech involves the speech motor system. Supporting a causal role of articulation in speech perception, temporary impairment of lip (vs. tongue) motor brain regions has been found to deteriorate, though not completely impair, discrimination of phonemes articulated with the lips (vs. tongue; D'Ausilio et al., 2009; for reviews of evidence for and against motor influences on speech perception, see Galantucci et al., 2006; Skipper et al., 2017).

Similar to speech, for music, there has also been evidence for bidirectional contributions of action and perception processes (e.g., Godøy et al., 2016; Zatorre et al., 2007; for reviews, see Keller, 2012; Maes et al., 2014; Novembre & Keller, 2014; Schiavio et al., 2014). For example, a manipulation of movement rhythm has been found to influence later judgments concerning heard rhythms (Phillips-Silver & Trainor, 2007). Moreover, musical imagery can be disrupted by concurrent auditory or articulatory tasks, so that participants made more mistakes when comparing a heard musical theme to a previously seen musical notation if they concurrently performed (vs. did not perform) a rhythmic or articulatory task (Brodsky et al., 2008; see also Brown & Palmer, 2013; for other motor influences, see Connell et al., 2013; Jakubowski et al., 2015). In sum, action has been argued to be involved in the perception and understanding of both speech and music.

Related predictions are made by embodied cognition theories. Embodied cognition postulates that information processing involves the simulation of sensory, motor, or emotional experiences (Barsalou, 2008; Kiefer & Pulvermüller, 2012; Winkielman et al., 2018; for reviews, see Glenberg, 2010; Körner et al., 2015; Körner et al., 2023; Meteyard et al., 2012). Interference with these sensorimotor simulations has been found to alter cognitive processing. Concerning the influence of facial posture, previous research concentrated on emotional content (Niedenthal, 2007). Specifically, emotional information was processed faster (Havas et al., 2007) and detected more accurately (Niedenthal et al., 2001, 2009) when participants’ facial expression could be congruent (e.g., smiling for positive information) compared to when it had to be incongruent (e.g., smiling for negative information) with the valence of the information. Moreover, participants’ mouth posture has been found to influence evaluations (Strack et al., 1988). When muscles involved in smiling were activated by holding a pen with their teeth (vs. protruding lips), participants rated cartoons to be funnier (Strack et al., 1988; for a meta-analysis, see Coles et al., 2019). Note, however, that the boundary conditions of this effect are still under debate (Wagenmakers et al., 2016; see also, Coles et al., 2022; Noah et al., 2018; Strack, 2016). From embodied cognition research, we predict that altering the posture of muscles involved in singing could influence the chant produced during singing imagery.

## The influence of mouth posture on pitch

Mouth posture can influence pitch via different pathways: first, as a direct link; second, through associations with fundamental and formant frequencies of vowels; third, through associations with valence. The direct influence of mouth posture on pitch could result from changed vocal tract length. Retraction of the lip corners shortens the vocal tract (Shor, 1978); conversely, rounding and protruding the lips lengthens the vocal tract (Dusan, 2007; Fant, 1960), which facilitates lower-pitched utterances (Ohala, 1984). Accordingly, in chimpanzees, greater (vs. lesser) lip retraction has been found to be associated with higher pitched vocal utterances (Bauer, 1987). Similarly, when human participants were asked to smile while speaking, their pitch increased (Barthel & Quené, 2015; Tartter, 1980; for the reverse influence, see Huron et al., 2009).

Second, facial muscle activity also influences the articulation of vowels; vowels, in turn, differ in their fundamental frequency (which is perceived as pitch) and formant frequencies. 1 For example, /i/ – the vowel with the greatest lip corner retraction – has a higher fundamental as well as second formant frequency than /o/ – a vowel with strong lip protrusion (Hoole & Mooshammer, 2002). Second formant frequency also influences subjective tone height, so that when the fundamental frequency is identical, vowels with low (vs. high) second formant are perceived as higher (Fowler & Brown, 1997; see also Russo et al., 2019). Consistent with both the relative fundamental and second formant frequencies of /i/ and /o/, yodelers (which consist largely of nonsense syllables) typically employ /o/ for low-pitched passages and /i/ for high-pitched passages (Fenk-Oczlon & Fenk, 2009). Thus, lip retraction (protrusion), by being associated with intrinsically high-pitched (low-pitched) vowels, could increase (decrease) pitch.

Third, the posture–pitch association might result from a common association with valence. High pitch and positive valence have been found to be associated on a conceptual level (Eitan & Timmers, 2010). Moreover, in various species, including humans, high pitch signals friendly, affiliative behavior, whereas low pitch signals aggressive, dominating behavior (Hodges-Simeon et al., 2010; Morton, 1977; Wood et al., 2017). Retracting (vs. protruding) lips might have been used to influence pitch to signal deference (vs. aggression; Ohala, 1984). Relatedly, vowels differ in their perceived valence. Specifically, /i/ has been found to be associated with positive valence and /o/ with negative valence (Rummer et al., 2014; see also Garrido & Godinho, 2021; Körner & Rummer, 2022b; Rummer & Schweppe, 2019; Yu et al., 2021). Moreover, this association between vowels and valence has been found to rest on articulation muscle activity (Körner & Rummer, 2022a). Thus, lip retraction (protrusion) could further increase pitch because positive (negative) valence is associated with high (low) pitch.

In sum, several processes might cause facial postures with retracted (vs. protruded) lips to be associated with high (vs. low) pitched vocalizations. In the present research, we manipulated participants’ facial posture and assessed pitch during singing imagery. Singing involves pitch changes relating to the melody as well as to the articulation of the lyrics. The vowels in lyrics differ in fundamental and formant frequencies. As described above, during singing, facial posture could alter pitch, either by itself or through its relation to vowel frequencies or vowel valence. Because covert articulation is instrumental in singing imagery, we hypothesized that facial posture would influence pitch even though participants only imagined singing.

## Experiment 1

Experiment 1 provides a first test regarding whether mouth posture influences pitch during singing imagery. Participants were asked to adopt a posture associated with the pronunciation of either /i/ or /o/ while singing mentally. We hypothesized that an i-posture compared to an o-posture would lead to higher pitch during singing imagery.

### Method

#### Transparency and openness

We report sample size determination, all data exclusions (if any), all manipulations, and all measures. All data, analysis code, and research materials are available via the Open Science Framework at https://osf.io/uzfq8/. Both experiments (hypothesis, design, and main analysis) were pre-registered; Experiment 1: https://osf.io/g96q4; Experiment 2: https://osf.io/q76hu.

#### Participants

We used *d* = 0.40 as the smallest effect size of interest. To detect *d* = 0.40 with 1-β = .90 and α = .05 in a one-tailed between-participants *t*-test, 216 participants are required. We pre-registered sequential testing (Lakens, 2014) with two interim analyses. Calculating the adjusted alpha-levels using *GroupSeq* (version 1.3.4; Pahl, 2018) with α*t^1^ as spending function resulted in the following *p*-values for rejecting the null-hypothesis: *p* < .017 with 72 participants, *p* < .023 with 144 participants, and *p* < .030 with 216 participants. The first interim analysis satisfied the stopping criterion, resulting in 72 participants (49 female, 23 male; 58 German native speakers; *M*_*age*_ = 26 years, *SD*_*age*_ = 6 years, recruited on a German university campus, without screening for musical training).

#### Mental singing task

Participants were informed that their task was to imagine singing parts of given songs; singing “in their heads” as clearly as possible while listening with their “inner ear,” but taking care not to sing aloud. For each song excerpt, participants first read the title. When they were ready to begin singing, they were shown the sheet music for the song excerpt with the lyrics underneath the music staff lines. Participants indicated both the beginning and end of their mental singing by key presses. After mentally singing the provided song excerpt, they evaluated the song and their mental singing on five dimensions (e.g., speed and clarity). Embedded was the dependent measure. Participants answered (translated) *How high did the song sound in your head (we refer to pitch)?* on a 7-point scale ranging from 1 (*very deep*) to 7 (*very high*). When all evaluations were completed, the next song followed.

There were two sets of songs, each consisting of one Christmas carol, two classic pop songs and one children’s song. Participants received one set during the baseline phase and the other during the experimental phase (counterbalanced across participants). Valence evaluations by an independent group of participants indicated that these songs were on average experienced to be mildly to moderately positive (see the Appendix in Online Supplemental Material (OSM)).

#### Procedure

Participants first provided informed consent. Then a baseline phase ensued, consisting of four singing imagery trials. For this, participants were asked to sing mentally without receiving instructions about facial posture. Each singing trial consisted of the mental singing phase, followed by the evaluation phase.

**Fig. 1:**
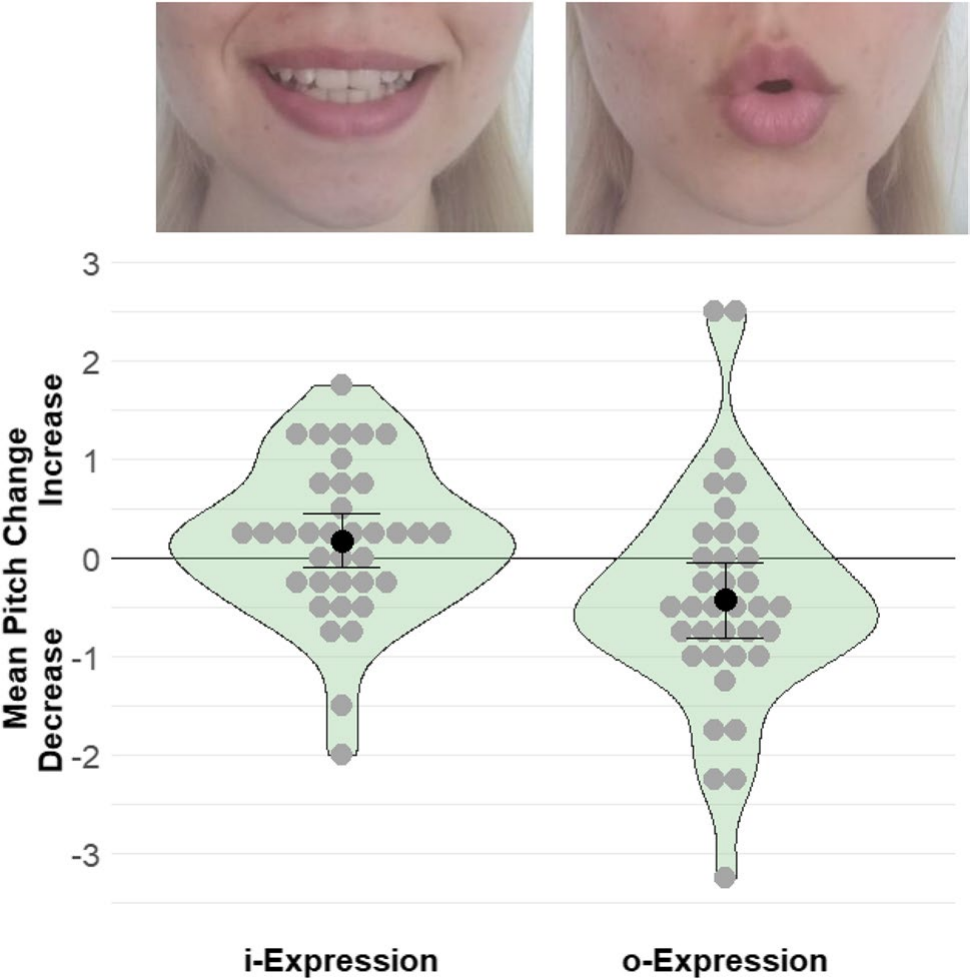
**Pi**ctures explaining the instructions for the facial posture (**upper panel**) and plot of the results (**lower panel**) of Experiment 1. The results show how pitch judgments changed from the baseline to the experimental block depending on mouth posture. *Note.* The black dots and error bars depict means and 95% confidence intervals, the gray dots depict individual participant means, and the shapes are density plots

Then the experimental phase ensued. Participants were randomly assigned to either the i-posture or the o-posture. They were asked to adopt a facial expression as if they were articulating the letter i (or o)^2^ for another four songs. To illustrate the mouth posture, participants were shown the lower part of a face adopting the desired pose; see Fig. 1 upper panel. Before each trial in the experimental phase, participants were asked to adopt the pose and maintain it during the mental singing. For the subsequent evaluations, participants could relax their face.

After they had mentally sung and evaluated all eight songs, participants were asked to provide demographic information and to guess the purpose of the manipulation.

### Results and discussion

The dependent measure was the mean pitch judgment in the experimental trials minus mean pitch judgment in the baseline trials. Accordingly, pitch values greater (lesser) than 0 indicate an increase (decrease) in pitch relative to baseline.

Confirming our hypothesis, pitch judgments were higher (*M* = 0.18, *SD* = 0.79) for participants who adopted an i-posture than for participants who adopted an o-posture (*M* = -0.42, *SD* = 1.14), *t*(62) = 2.62, *p* = .006 (one-tailed), *d* = 0.62, 95% CI [0.14; 1.09], see Fig. 1.^3^ Exploratory analyses indicated that, relative to the baseline, adopting an o-posture decreased pitch judgments, *t*(35) = 2.23, *p* = .032, *d*_z_ = 0.37, 95% CI [0.03; 0.71], while adopting an i-posture did not significantly increase pitch judgments, *t*(35) = 1.38, *p* = .178, *d*_z_ = 0.23, 95% CI [-0.10; 0.56].

Thus, Experiment 1 provides first evidence that articulation posture influences the pitch of singing imagery, with increased pitch for an i-posture compared to an o-posture. However, it cannot be excluded that this effect was caused by participants’ thinking about the articulation of /i/ or /o/. To preclude this possibility, the manipulation in Experiment 2 did not refer to vowel articulation.

## Experiment 2

Experiment 2 employed a slightly different manipulation of articulation posture – participants held a straw in their mouth similar to Strack et al. (1988). We expected to replicate the results of Experiment 1 even when participants were not led to think of their facial posture in terms of vowel articulation.

### Method

#### Participants

We again used sequential testing, this time with one intermediate test after about 55% of the maximum target *N* of 150 (resulting from a power analysis with *d* = 0.41^4^, 1-β = .80, and α = .05 for the one-tailed between-participants *t*-test). The adjusted alpha-levels were *p* < .028 with about 85 participants and *p* < .034 with 150 participants. This resulted in 88 participants (56 female, 32 male; 83 German native speakers; *M*_age_ = 29 years, *SD*_age_ = 10 years; recruited from a local participant pool of a German university that mainly consists of undergraduates; they were not screened for musical training).

#### Procedure

**Fig. 2:**
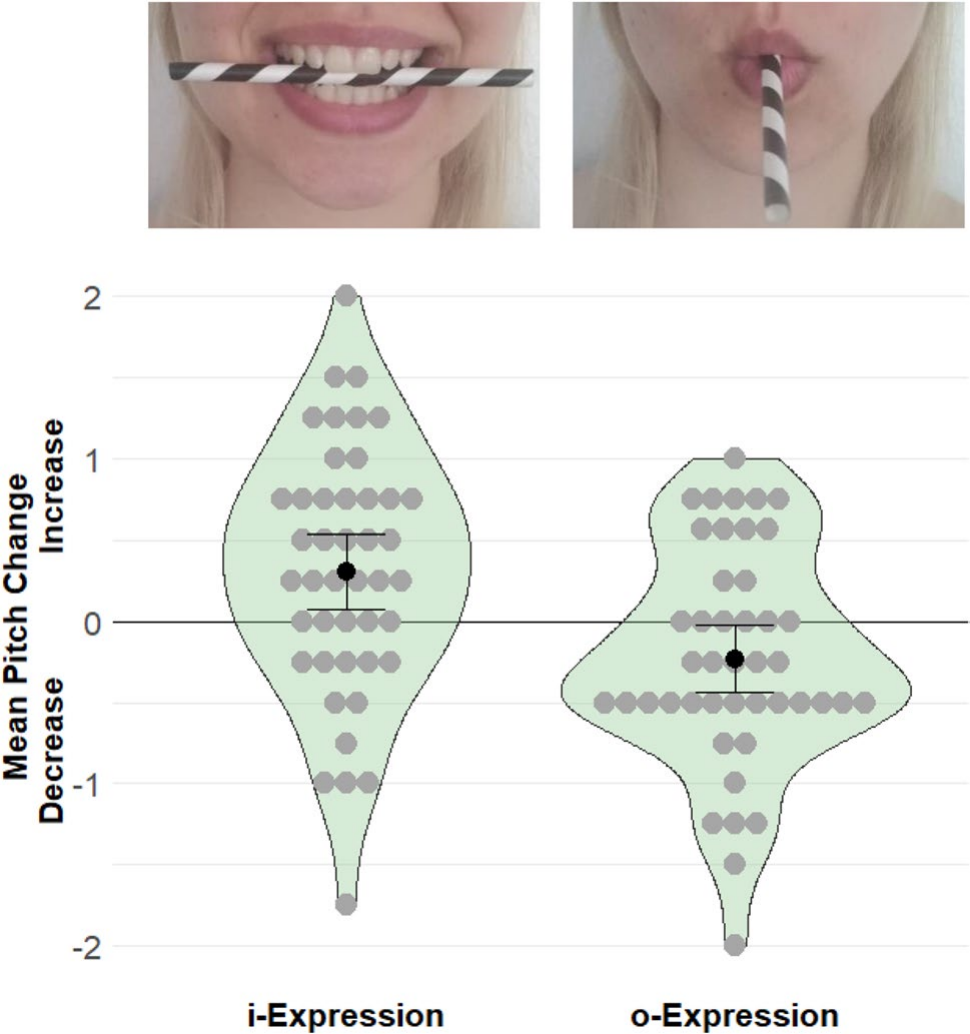
Pictures explaining the instructions for the facial posture (**upper panel**) and plot depicting the results (**lower panel**) of Experiment 2. The results show how pitch judgments changed from the baseline to the experimental block depending on mouth posture. *Note.* The black dots and whiskers depict means and confidence intervals, the gray dots depict individual participant means, and the shapes are density plots

Materials and procedure were identical to Experiment 1 except for minor wording changes and the facial manipulation. After the baseline trials, participants were asked to hold a straw in their mouth to control facial movements during mental singing. Half the participants were instructed to hold the straw vertically using their teeth while avoiding their lips touching the straw. The remaining participants were instructed to hold the straw with their protruded lips while avoiding their teeth touching the straw. To ensure correct execution, the corresponding picture of Fig. 2, upper panel, was shown. For the evaluation phase, participants were asked to remove the straw, and afterwards they were reminded to replace it between their teeth/lips.

### Results and discussion

As in Experiment 1, the pitch judgments were significantly higher for participants who adopted an i-posture (*M* = 0.31, *SD* = 0.77) than for participants who adopted an o-posture (*M* = -0.23, *SD* = 0.67), *t*(84) = 3.51, *p* < .001 (one-tailed), *d* = 0.75, 95% CI [0.31; 1.18], see Fig. 2.^5^ Exploratory analyses indicated that, compared to baseline, adopting an o-posture decreased pitch judgments, *t*(43) = 2.31, *p* = .026, *d*_z_ = 0.35, 95% CI [0.04; 0.65], while adopting an i-posture increased pitch judgments, *t*(43) = 2.64, *p* = .012, *d*_z_ = 0.40, 95% CI [0.09; 0.70].

Thus, Experiment 2 replicates the results of Experiment 1. A posture resembling the articulation of /i/ resulted in higher pitch judgments than a posture resembling the articulation of /o/.

## General discussion

The present work examined whether mouth posture alters pitch during singing imagery. In two experiments, we found that relative to baseline, participants who retracted their lips in a manner similar to the articulation of /i/ experienced higher pitch during singing imagery and participants who protruded their lips in a manner similar to the articulation of /o/ experienced lower pitch during singing imagery. This influence occurred both when participants were instructed to shape their mouth as if to articulate /i/ (vs. /o/; Experiment 1) and when their mouth posture was manipulated by holding a straw (Experiment 2). Thus, mouth posture influenced pitch in singing imagery.

In the present research, our sample was not selected for musical proficiency. Musical training has been found to improve musical imagery ability, especially concerning pitch (Aleman et al., 2000; Janata & Paroo, 2006). Therefore, we would expect even clearer and more accurate pitch imagery for musical experts, so that the effect of mouth posture on pitch could be stronger for musicians than for non-musicians. However, trained singers need to be able to independently modulate mouth posture for articulation and pitch. Accordingly, for singers, mouth posture and pitch might be decoupled, so that the effect of mouth posture on pitch might be reduced. Whether this is indeed the case needs to be determined in future research.

Another open question is whether mouth posture influences pitch *production* (i.e., the inner voice) or pitch *perception* (i.e., the inner ear). If, in accordance with the above presented potential mechanisms (oral tract length, vowel association, or valence association) mouth posture influences the inner voice, then overt recreation of the acoustic frequency produced by the inner voice should differ depending on mouth posture. However, if mouth posture influences the inner ear, then the objective frequencies of the mental singing might be identical; but hearing the same frequency would lead to differing sensations of pitch depending on mouth posture (for a related finding, see Hostetter et al., 2019). Future research needs to determine whether mouth posture distorts the inner voice or the inner ear.

Strictly speaking, we cannot rule out a third possibility, namely that mouth posture influences pitch only retrospectively. However, the repeated nature of the task renders this possibility unlikely. After a few trials of the baseline block, participants probably remember the questions and form their judgments already during the singing imagery. Moreover, in the experimental block, participants were asked to relax their face during judgments. Thus, any influence during the judgment phase should have weakened the influence of mouth posture because mouth posture did not differ anymore between the conditions. Nevertheless, future research may rule out the contribution of post-singing processes on pitch judgments by asking participants to evaluate pitch during the singing period.

A possible moderator for the present finding is song valence. Facial muscle activity is involved not only in articulation and pitch production but also in emotional processes. An exploratory correlation between song valence and effect size suggests that songs that are more positive might have larger effect sizes; however, this correlation was not significant (see OSM). In the present research, all employed songs were positive. Future research needs to examine whether the influence of facial posture on pitch also holds for negative songs or conversely only obtains for positive songs.

By finding that mouth posture influences pitch during singing imagery, the present research contributes to the literature on language–music similarities (Nayak et al., 2022; Patel, 2010). Although it is clear that the cognitive processing of speech and music is not identical (e.g., Ilie & Thompson, 2006; Zatorre et al., 2002), various parallels and mutual influences have been observed (Canette et al., 2020; Coffey et al., 2017; Deutsch et al., 2011; Gordon et al., 2015; Slevc et al., 2009; Thompson et al., 2004; Weiss & Trehub, 2022; Yang et al., 2022). Pitch changes in music and language, for example, have been found to be processed by overlapping brain regions (Schön et al., 2010) and to share mechanisms, so that, for example, speaking a tone language is associated with enhanced musical pitch perception (Bidelman et al., 2013; Giuliano et al., 2011; Pfordresher & Brown, 2009). The present research also speaks to a connection between language and music by demonstrating that adopting a mouth posture for vowel articulation influences pitch perception in music imagery. Thus, complementing previous research that mainly examined individual differences or long-term training effects, we find that a situational manipulation of the articulation posture influences the experiential nature of music perception.

### Supplementary information

ESM 1 (DOCX 59 kb)

## Data Availability

All data and materials are available at https://osf.io/uzfq8.

## Footnotes

1 Formants are energy peaks in the frequency spectrum. In speech, formants distinguish phonemes; the first and second formants are frequently sufficient to distinguish vowels.

2 As German has a close grapheme to phoneme mapping, the letter graphemes indicated which phonemes were meant.

3 An alternative analysis, incorporating random effects for both participants and stimuli and treating responses as ordinal responses generated by a latent continuous scale, yields similar results, see the OSM.

4 As sequential testing can lead to overestimated effect sizes (Lakens, 2014), we used two-thirds of the effect size observed in Experiment 1.

5 As in Experiment 1, an ordinal mixed effects analysis yields similar results, see OSM. Additionally, the OSM also includes a correlation analysis indicating that increasingly positive song valence might be associated with increasing effect sizes.

## Code availability

All code is available at https://osf.io/uzfq8.
