# Supplementary material for: Articulation posture influences pitch during singing imagery
Source: Psychon Bull Rev. 2023 May 23;30(6):2187–95. doi: 10.3758/s13423-023-02306-1 (PMC10728233; doi:10.3758/s13423-023-02306-1)
Supplement: Supplementary file 1 — (DOCX 59 kb) [file 13423_2023_2306_MOESM1_ESM.docx]

Ancillary Analyses for „**Articulation Posture Influences Pitch During Singing Imagery”**

**Testing the Influence of Posture on Pitch Using Ordinal Mixed Effects Models**

In the main text, we treated pitch evaluations on Likert-type scales as interval-scaled data and accordingly calculated means and other statistics required for the pre-registered t-tests. However, it has been argued that Likert-type responses should always be treated as ordinal (e.g., Liddle & Kruschke, 2018). Moreover, given that there were multiple responses per participant, mixed effect models are appropriate. Therefore, as an alternative to the t-tests reported in the main text, we analyzed the data using ordered logistic mixed effects models with crossed random effects for participants and stimuli (see Bürkner & Vuorre, 2019) using the R package brms (Bürkner, 2017). The responses were modelled as thresholds set on a latent continuous scale with a cumulative likelihood function and a logit link function using uninformative priors. Block (baseline vs. experimental; within-participants) and posture (i-posture vs. o-posture; between-participants) as well as their interaction were entered as fixed factors in addition to a full random effects structure (random intercepts and random slopes for participants and stimuli). In both Experiments, the posterior credibility intervals for the interaction did not include 0, see Tables S1 and S2. This indicates that the interaction of block and posture significantly influenced pitch judgments. Thus, the main results of these analyses are consistent with the analyses reported in the main text. Figures S1–S4 give an impression of the posterior probabilities of response options depended on experimental condition; for more details, see https://osf.io/my57h, p. 9­–16 (for Experiment 1) and p. 24–31 (for Experiment 2).

**Table S1**

*Estimates, Standard Errors, and 95% Posterior Credibility Intervals for the Estimates for Experiment 1. The Model Depicts the Influence of Block (Baseline vs. Experimental) and Posture (i-posture vs. o-posture) on Pitch Judgments.*

| Parameter | Posterior parameter mean | Standard error of the posterior parameter | 95% credible interval |
| --- | --- | --- | --- |
| Threshold 1 | -5.19 | 0.54 | [-6.28; -4.19] |
| Threshold 2 | -2.30 | 0.36 | [-3.03; -1.63] |
| Threshold 3 | -0.71 | 0.34 | [-1.39; -0.05] |
| Threshold 4 | 0.48 | 0.33 | [-0.18; 1.15] |
| Threshold 5 | 2.39 | 0.36 | [1.68; 3.13] |
| Threshold 6 | 4.39 | 0.46 | [3.52; 5.33] |
| Block | 0.31 | 0.35 | [-0.37; 1.00] |
| Posture | 0.20 | 0.26 | [-0.32; 0.71] |
| Interaction Block and Posture | -0.97 | 0.40 | [-1.78; -0.19] |

**Table S2**

*Estimates, Standard Errors, and 95% Posterior Credibility Intervals for the Estimates for Experiment 2. The Model Depicts the Influence of Block (Baseline vs. Experimental) and Posture (i-posture vs. o-posture) on Pitch Judgments.*

| Parameter | Posterior parameter mean | Standard error of the posterior parameter | 95% credible interval |
| --- | --- | --- | --- |
| Threshold 1 | -5.12 | 0.54 | [-6.25; -4.11] |
| Threshold 2 | -2.73 | 0.34 | [-3.38; -2.07] |
| Threshold 3 | -0.72 | 0.31 | [-1.32; -0.12] |
| Threshold 4 | 0.46 | 0.31 | [-0.14; 1.07] |
| Threshold 5 | 2.50 | 0.33 | [1.88; 3.15] |
| Threshold 6 | 4.43 | 0.41 | [3.64; 5.25] |
| Block | 0.54 | 0.23 | [0.09; 0.98] |
| Posture | 0.42 | 0.24 | [-0.06; 0.90] |
| Interaction Block and Posture | -1.01 | 0.29 | [-1.58; -0.45] |

**Figure S1**

*Marginal Effects of o-Posture on Pitch Judgments in Experiment 1. Dots Depict the Posterior Mean Estimates of Each Response Option by Participants in the o-Posture Condition, for the Baseline and the Experimental Block Separately. Error Bars Are 95% Credible Intervals.*


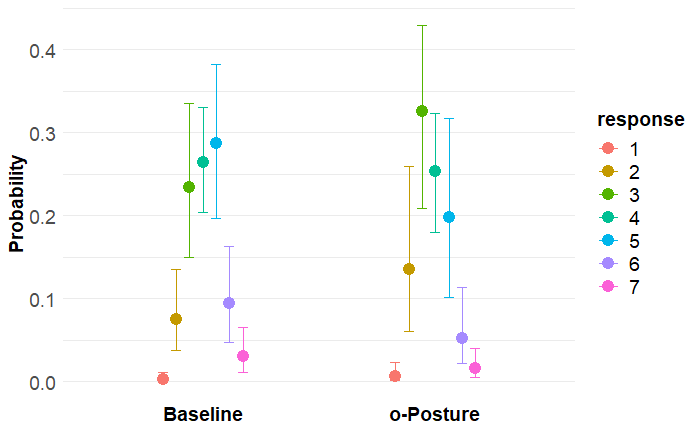


**Figure S2**

*Marginal Effects of i-Posture on Pitch Judgments in Experiment 1. Dots Depict the Posterior Mean Estimates of Each Response Option by Participants in the o-Posture Condition, for the Baseline and the Experimental Block Separately. Error Bars Are 95% Credible Intervals.*


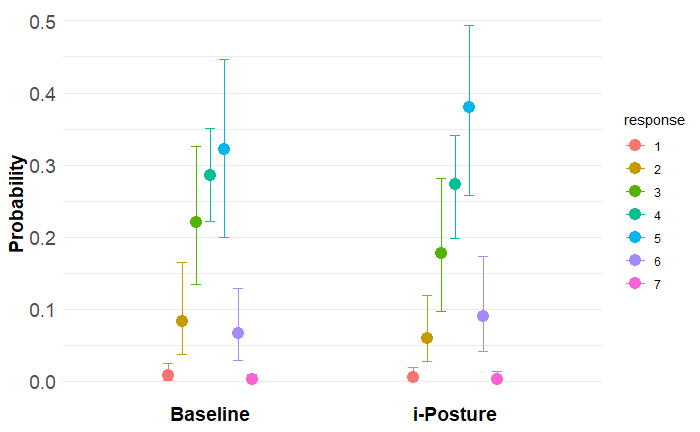


**Figure S3**

*Marginal Effects of o-Posture on Pitch Judgments in Experiment 2. Dots Depict the Posterior Mean Estimates of Each Response Option by Participants in the o-Posture Condition, for the Baseline and the Experimental Block Separately. Error Bars Are 95% Credible Intervals.*


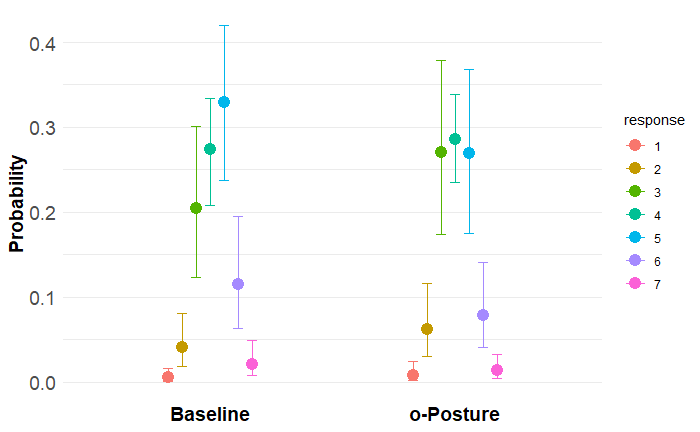


**Figure S4**

*Marginal Effects of i-Posture on Pitch Judgments in Experiment 2. Dots Depict the Posterior Mean Estimates of Each Response Option by Participants in the o-Posture Condition, for the Baseline and the Experimental Block Separately. Error Bars Are 95% Credible Intervals.*


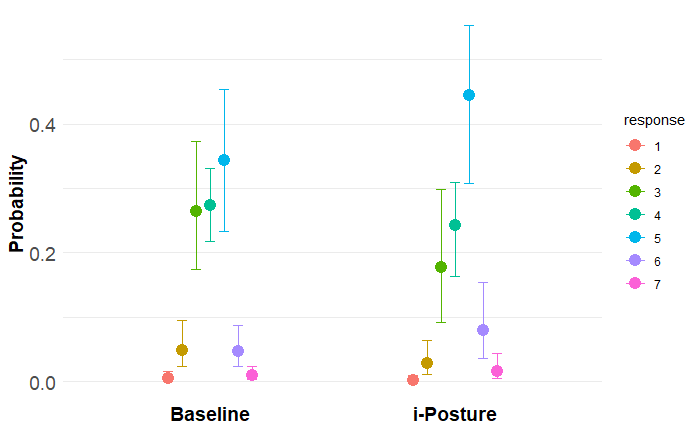


**Does the Effect (Influence of Posture on Pitch) Correlate With Song Valence?**

To examine whether the influence of the manipulation varied with song valence, we correlated effect sizes with mean valence evaluations for each song. To determine song valence, 42 participants who had not participated in either Experiment 1 or Experiment 2 were recruited. Participants received the same sheet music and were asked to perform the singing imagery task. After each song, they were asked *How does singing this song influence your mood?* (translated). Responses were made on a 7-point scale (1 = *very negatively* to 7 = *very positively*). The evaluations for each song were averaged across all participants leading to a mean evaluation score for each song.

Mean effect sizes were calculated as standardized mean difference scores (Cohen’s *d*) for each song. Specifically, for each song, data from the experimental blocks of both experiments were combined. Mean evaluations by participants in the i-condition minus mean evaluation by participants in the o-condition were calculated (so that positive effect sizes indicate that the pitch was higher for the i-condition than for the o-condition, which was the case for all songs) and divided by the pooled standard deviation.

The rank correlation between effect size and mean song evaluation did not reach significance, Kendall’s τ = .429, *p* = .179, see Figure S1. Although not statistically supported in the present study, the effect size (and therefore the influence of posture on pitch judgments) might increase with increasing song valence. To determine whether this is the case, a higher number of songs with more varied valence needs to be examined to acchieve sufficient power.

**Figure S5**

*Correlation between Song Valence Evaluations and Observed Effect Size*


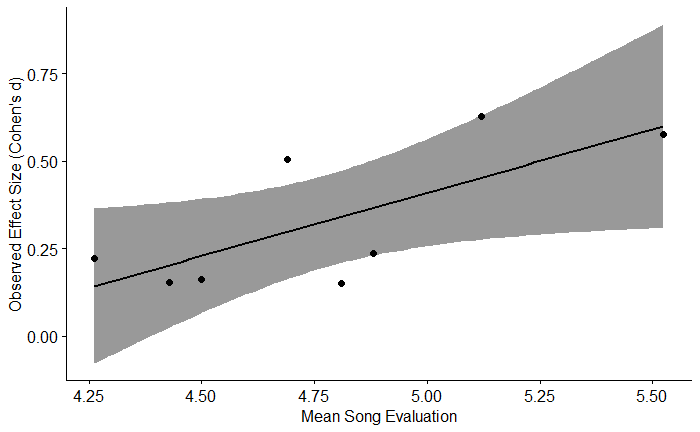


*Note.* Dots depict individual songs. The line represents a regression line with confidence bands. Song evaluation ranged from 1 (very negative) to 7 (very positive). (Increasingly) positive effect sizes indicate that mean pitch evaluation in the experimental block was (increasingly) higher for participants with an i-expression compared to an o-expression.

**Does the Effect Change Over the Course of the Experimental Block?**

If the emotional meaning of the facial posture influences pitch judgments, one could expect this influence to increase over the course of the experimental block. Therefore, we examined the influence of mouth posture for the first half and the second half of the experimental block separately. To increase power (to compensate for the loss of the baseline trials), we combined the data from both experiments. Pitch judgments were entered into a facial posture (i-posture vs. o-posture; between) X order (first half vs. second half; within) mixed model ANOVA. Neither the main effect of order, *F*(1, 158) = 2.19, *p* = .141, $\eta_{p}^{2}$ = .014, 95% CI = [.000; .058], nor the interaction between order and posture reached significance, *F*(1, 158) = 0.17, *p* = .677, $\eta_{p}^{2}$ = .001, 95% CI = [.000; .024]. However, the main effect of posture was significant, *F*(1, 158) = 10.29, *p* = .002, $\eta_{p}^{2}$ = .061 95% CI = [.015; .131], consistent with the main analysis that i-posture leads to higher pitch judgments than o-posture. For more details and additional analyses, see <https://osf.io/my57h>, p. 37–39. Thus, in the present experiment, there is no evidence that the influence of posture on pitch changed over time.

**References**

Bürkner, P.-C. (2017). brms: An R package for Bayesian multilevel models using Stan. *Journal of Statistical Software, 80*(1), 1–28. https://doi.org/10.18637/jss.v080.i01

Bürkner, P.-C., & Vuorre, M. (2019). Ordinal regression models in psychology: A tutorial. *Advances in Methods and Practices in Psychological Science*, *2*(1), 77–101. https://doi.org/10.1177/2515245918823199

Liddell, T. M., & Kruschke, J. K. (2018). Analyzing ordinal data with metric models: What could possibly go wrong? *Journal of Experimental Social Psychology, 79*, 328–348. https://doi.org/10.1016/j.jesp.2018.08.009
